# Determinants of Familiarity and Experience with HIV Pre-Exposure Prophylaxis in Primary Care Providers in Ontario, Canada

**DOI:** 10.1177/21501319251315566

**Published:** 2025-01-23

**Authors:** Jorge Martinez-Cajas, Beatriz Alvarado, Carmela Rapino, Emma Nagy, T. Hugh Guan, Nicholas Cofie, Nancy Dalgarno, Pilar Camargo, Bradley Stoner

**Affiliations:** 1 Division of Infectious Diseases, Department of Medicine, Queen’s University, Kingston, Canada; 2 Department of Public Health Sciences, Queen’s University, Kingston, Canada; 3 Kingston, Frontenac, Lennox & Addington Public Health Unit; 4 Office of Professional Development and Educational Scholarship, Queen’s University, Kingston, Canada; 5 School of Nursing, Queen’s University, Kingston, Canada

**Keywords:** implementation science, HIV pre-exposure prophylaxis (PrEP), HIV prevention, primary care providers (PCPs)

## Abstract

**Background:**

Despite increased access to HIV pre-exposure prophylaxis (PrEP) in Canada, familiarity and experience among primary care providers (PCPs)—including family doctors and those working with key populations—remains limited. To understand the barriers and facilitators of PrEP familiarity and experience, we conducted a situational analysis in PCPs in sub-urban and rural Ontario.

**Methods:**

We surveyed a non-probabilistic sample of PCPs using an online questionnaire, designed with the Consolidated Framework for Implementation Research (CFIR). Poisson regressions with robust variance were used to assess the relationship between CFIR domains, sociodemographic, and practice characteristics on both PrEP familiarity and experience.

**Results:**

A total of 54 PCPs participated (6% response rate), comprising 80% physicians and 20% nurses. Nearly 30% of the sample worked with key populations, including sexual health clinics and community care centers, 18% of respondents reported high familiarity with PrEP, and 44% reported PrEP experience (referred, started a conversation, or prescribed). PrEP familiarity and experience were associated with working in an organization serving key populations, working with gender minorities, and having colleagues providing PrEP. Providers with a positive perception of PrEP and its necessity for populations at risk were more likely to have PrEP-related experience. Higher familiarity and experience were reported by PCPs with specific clinical skills related to PrEP, and with the perception that PrEP was compatible with their practice as primary provider.

**Conclusions:**

Our findings suggest that organizational support, and additional training and education would facilitate PrEP provision by PCPs in suburban/rural Ontario.

## Introduction

HIV Pre-Exposure Prophylaxis (PrEP) is a key medical intervention to end the HIV epidemic, as it can reduce the risk of HIV acquisition by 86% in highly adherent individuals at high risk of HIV.^1^ Besides, oral PrEP has been shown to contribute to unprecedented reductions in HIV incidence in various settings.^2-4^ In Canada, the publication of national PrEP guidelines^5^ was followed by a steep increase in PrEP use, yet to date it remains underutilized.^6^ A survey in the three largest Canadian cities between 2017 and 2019 estimated that less than 25% of PrEP-eligible Gay Bisexual Men who Have Sex with Men (GBM) had taken PrEP within the previous 6 months.^7-9^ PrEP uptake is even lower among cisgender women and men, people who reside in rural or suburban areas, and in provinces with a high proportion of Indigenous populations.^10-13^ The introduction of PrEP promotion programs and policy initiatives to cover PrEP,^9^ and shifting PrEP provision to nurse-lead programs in some localities^14,15^ have had some success. Nevertheless, there continues to be a critical need to increase PrEP awareness, uptake, and provision, especially outside large urban centres.^7,12,16,17^

Several barriers have been identified that limit the implementation of PrEP services. Population groups at high risk of HIV acquisition face barriers such as lack of health insurance, PrEP and HIV-related stigma, mistrust, reluctance to consult primary care providers (PCPs), and fear of adverse effects, among others.^10,17-20^ Provider-level barriers are related to the PCPs’ unwillingness or inability to offer PrEP,^21-26^ and attitudes and beliefs towards PrEP.^27^ Contextual barriers relate to affordability and availability of and access to PrEP services that are determined by the way the health system is organized and funded,^28^ and the disruption of sexual health programs by the COVID-19 pandemic.^29^ In Canada, the leading PrEP uptake barriers in people at risk of HIV acquisition are cost, concern about side effects of PrEP medications, and underestimation of HIV acquisition risk.^7,30,31^

Physicians and nurse practitioners in Canada have traditionally prescribed oral PrEP and monitor their users longitudinally. Nurses and community organizations have had a less active role in PrEP provision, although discussions on increasing their involvement have taken place.^32^ Published reports indicate that 47% of PrEP prescriptions in Canada are written by primary care physicians, whereas 16% are written by specialist physicians, and 37% by other practitioners (e.g., nurse practitioners, physician specialty unknown).^16^ A similar trend has been observed in Ontario, where 36.5% of the PrEP prescriptions are issued by family physicians, 19% by specialists, and 40.9% by physicians of unknown specialty.^33^ The vast majority of PrEP dispensing in Ontario is concentrated in large cities (in 2023, 75.4% of PrEP prescriptions in Ontario were dispensed in Toronto and Ottawa).^34^ In Ontario, a large infrastructure of sexual health clinics, managed by public health units, are the first point of contact for many individuals seeking medical care for sexually transmitted infections.^35^ Notably, a very small proportion of them offer PrEP services.^36^ Online providers have also contributed to the increase of PrEP prescription in Ontario, however, overall adherence to and persistence on PrEP remain unknown.

In Canada, several studies have reported on the barriers PCPs face that interfere with PrEP adoption including lack of training, lack of motivation, organizational aspects of PCP’s practices, and low compatibility of PrEP with their work.^27,28,37^ These studies assessed PCPs’ willingness to prescribe PrEP before national guidelines were published, were conducted in large urban centers, and did not represent PCPs working with special populations and in public health settings, namely sexual health clinics.

In response to the above, we gathered data from PCPs across various settings in Ontario that have remained underrepresented in PrEP implementation research with the following objectives: (1) to determine the level of PrEP familiarity and experience of PCPs working in suburban and rural practice settings, and (2) identify the main determinants of PrEP familiarity and experience, taking into consideration sociodemographic, practice-level, individual, and contextual factors. These factors are organized under a robust implementation science framework to guide the selection of strategies that could enhance future PrEP implementation in such settings.

## Data and Methods

### Study Design and Context

The analysis presented here is part of the primary objectives of the PrEP-SEO study. The PrEP-SEO study used a mixed-methods implementation research design, including online surveys and semi-structured interviews, to explore the determinants related to PrEP adoption by primary care providers and propose implementation strategies. This study initially recruited PCPs in Southeastern Ontario, whose population (approx. 600,000 inhabitants) mainly reside in small to mid-sized cities, suburban, and rural settings. The study was later extended to include PCPs serving other suburban areas in Ontario. In Southeastern Ontario, PrEP has been offered by a few primary care providers (PCPs), one subspecialty clinic, and one sexual health clinic. Most sexual health clinics and sites serving key populations in Ontario regions do not offer PrEP services. This study was conducted by a multidisciplinary team of public health, infectious diseases and primary care practitioners who were early PrEP prescribers.

### Study Participants and Recruitment

We invited healthcare providers from local primary care offices, community health centers, university student wellness centers and sexual health clinics who could prescribe, counsel, or screen potential PrEP users. We invited 941 PCPs practicing in Southeastern Ontario whose contact information was publicly available via the College of Physicians and Surgeons of Ontario and the Nurse Practitioners Association of Ontario websites. Survey invitations were faxed and mailed to all of them. Additionally, we invited 60 PCPs who had previously referred patients to a local sexual health clinic and 20 managers of sexual health clinics. Ninety-four providers accessed the survey, 36 answered no questions, and 4 completed less than 50%, leaving 55 with analyzable data.

### Measurements

*Outcomes*: The measured outcomes included: (i) Familiarity with PrEP, assessed as a Likert scale from 0 (not at all familiar) to 4 (extremely familiar); and (ii) Participants’ PrEP experience defined based on three questions assessing if, within the last 12 months, PCPs have: (1) *ever started a conversation, (2) prescribed PrEP to a client, or (3) referredpatients to PrEP providers*. If participants were not familiar with PrEP, their experience was considered absent.

*Demographics and practice characteristics*: We inquired about PCPs’ age, gender, minority status (any of the following: a self-perception of being a sexual, religious, or racial minority), years in practice, practice size, type of practice, location of practice, and characteristics of practice in terms of proportion of PCP’ patients who are sexual minorities, and people who use drugs. See the questionnaire in the supplemental file for details.

*Determinants related to the Consolidated Framework for Implementation Research (CFIR) framework*: We used the CFIR to identify determinants of PrEP familiarity and prescription.^38,39^ This framework, widely used in implementation science research in HIV, allows us to identify factors related to perceptions about PrEP, contextual (organizational and community), and individual factors related to familiarity and experience with PrEP, as supported by other work.^21,40,41^ The CFIR guided the development of statements that were extracted from previous studies on PrEP.^41 ^42^^ These statements covered 4 CFIR domains and were reviewed by 5 team members for relevance and clarity. This questionnaire was uploaded to Qualtrics for functionality assessment and underwent two rounds of feedback. One of the 5 CFIR domains, related to the planning of activities, was excluded from statement development as it lacked pertinence.

The remainder of the CFIR domains were included as follows: (i) 10 statements on PrEP characteristics (efficacy, advantage, simplicity), (ii) 23 statements on outer setting (attitudes toward population needs, concerns about PrEP use), (iii) 15 statements on inner setting (compatibility, organizational climate, resources, leadership engagement), and (iv) 30 statements on individual characteristics (comfort with clinical practices, beliefs about capabilities, professional role compatibility, beliefs about consequences). Table 1 presents the list of CIFR domains and their definitions. Table S1 to Table S9 show details on each statement and the derived scales.

**Table 1.** Summary of the Statements That Composed Each of the 4 Domains of CFIR Asked to Participants.

CFIR domain/subdomain	Description	Operationalization	Internal reliability—Alpha Chronbach
1. Characteristics of PrEP	Key attributes of PrEP that influence the success of its implementation		
Table S1	A total of 10 items assessed the perceptions of participants concerning characteristics of PrEP (Table 1s): effectiveness of the intervention (a1, a4), strength of evidence (a3), relative advantage (a5, a6, a7, a9), complexity (a10), cost (a8), and design quality (a2). Three items, a2, a5, and a7 were built as negative statements.	Likert scale from strongly disagree (value = 1) to strongly agree (value = 5), being reversed for negative values; therefore the higher score the higher positive attitudes toward PrEP characteristics	.72
2. Outer setting	The outer setting assessed two aspects, one was the acknowledgment of the need, resources, and benefits of PrEP in their patients, and the other related to concerns expressed by participants about the use of PrEP in their patients.		
2a. Perceptions on the interest/need of PrEP by patients or populations at risk Table S1	13 items included perceptions on the interest in PrEP by patients (p3, p5, p7, p10, p11), the affordability of PrEP (p2), the need for PrEP (p1, p4), the support of the community (p9), the adaptability of PrEP in their population (p8, p12) and the feasibility of using PrEP in the clinic (p6, p13). P1, p3, p4, p5 were worded as negative statements. Table 2s	Likert scale from strongly disagree (value = 1) to strongly agree (value = 5), being reversed for negative values; therefore the higher the score the more positive perception of the need in populations	0.80
2b. Concerns about the use of PrEP Table S2	10 statements that relate to accessibility, cost, increase in STIs, decrease in condom use, stigma, and misuse of medications.	Each statement takes a value between 0 to 10, The higher the value the higher the concern.	.80
3. Inner settings Table S3	Defines aspects of the organizations that may either facilitate or hinder the adoption of PrEP. In this domain, we explored 4 subdomains: compatibility, available resources, leadership engagement and organizational climate		.68
3a. Compatibility	Four items make up this subdomain: the readiness of the organization (r1), compatibility of PrEP with the organization (r5) and the perceptions of participants about the appropriate place for PrEP delivery (r3,r4).	Likert scale from strongly disagree (value = 1) to strongly agree (value = 5), being reversed for negative values; therefore the higher the score the higher the perception of compatibility.	
3b organizational climate	This includes 3 statements on the approval of colleagues to use PrEP (r2), orientation toward prevention (r6), and collaboration of professionals to deliver PrEP ( r7).	Likert scale from strongly disagree (value = 1) to strongly agree (value = 5), being reversed for negative values; therefore the higher the score the more positive perception of organizational climate	
3c. available resources	Five items assess the available resources for PrEP: enough patients interested in using PrEP (r8), sufficiency of time to deliver PrEP (r9), overall resources (r10, r11), and staff resources (r12).	Likert scale from strongly disagree (value = 1) to strongly agree (value = 5), being reversed for negative values; therefore the higher the score the more positive perception of available resources	
3d. leadership engagement	The last series of items relates to leadership engagement, and includes innovation (r13), sharing decisions (r14) and patient involvement (15)	Likert scale from strongly disagree (value = 1) to strongly agree (value = 5), being reversed for negative values; therefore the higher the score the more positive the perception of leadership engagement	
4. Individual characteristics	PCPs characteristics, such as the attitudes, beliefs, and skills, that might influence the adoption of PrEP. Four aspects were assessed under this CIFR domain		
4a. Comfort with clinical practice skills. Table S4	Level of comfort with nine clinical practice skills that could be necessary to adopt PrEP, such as inquiring about sexual orientation, discussing sexual habits, ordering a diagnostic test for HIV and other STIs, etc.	Likert scale from completely comfortable (value = 5) to completely uncomfortable (value = 1), the higher the value the more comfortable	.87
4b. Self-efficacy or ability to perform PrEP related practices Table S5	Self-perception of the capacity to do specific PrEP-related activities: offering complete PrEP care, counseling for PrEP, using tools to identify eligible patients, offering PrEP with a protocol, and manage effects of medications.	Each statement takes a value between 0 to 10, The higher the value the higher the ability or the self-efficacy.	.91
4c. Professional roles/ compatibility Table S6	These items included perceptions about fit with values (b1, b4), fit with clinical work (b2, b3), compatibility with role (b5, b7), worthiness (b6, b8), and priority (b9). Items b8 and b9 were negative statements.	Likert scale from strongly disagree (value = 1) to strongly agree (value = 5), being reversed for negative values; therefore the higher the score the more positive perception of professional compatibility	.88
4d. Beliefs about consequences Table S6	8 items assessed beliefs about consequences, which included, having extra monetary incentive (b10), gaining recognition (b11, b13, b15, b17) helping patients (b12 ), and strengthening relationships with colleagues (b14, b16).	Likert scale from strongly disagree (value = 1) to strongly agree (value = 5), being reversed for negative values; therefore the higher the score the more positive beliefs about consequences	.75

### Data Analysis

We conducted a descriptive analysis for CFIR-related items to explore data distribution and sample variability. We used non-parametric tests to compare PrEP familiarity and experience across demographic and practice characteristics. Then, we evaluated univariate relationships between each CFIR-related item and outcome using confidence interval-based estimation (CIBER) analysis implemented in R through the Jamovi platform.^43^ The CIBER analysis allows us to graphically observe the distribution of items and the bivariate relations with the study outcomes. With CIBER analysis, items with a more skewed distribution and association with outcomes are usually selected as targets for intervention.

The CFIR items were evaluated for internal consistency and then combined into scales representing each domain or subdomain (details provided in Table 1). These scales were generated by calculating the average score of each statement within the domain. The internal consistency of the scales ranged from 0.68 for the “Inner Setting” domain to 0.91 for “Self-Efficacy.” Due to a small sample size, single items were not included in the multivariate analysis as standalone variables.

A modified Poisson regression with robust variance was used to obtain the prevalence ratio (PR) which is a measure that approximates a risk ratio in cross-sectional studies.^44^ One model was built for familiarity (1 = extremely/very/moderately/familiar vs 0 = not at all/slightly familiar) and another for PrEP experience (1 = any of the following in the last 12 months, prescription, referral, or initiation of a conversation vs 0 = none of them). AIC and BIC criteria were used to define the best model explaining familiarity and PrEP experience. This analysis was done in STATA.

## Results

### Description of Participants

The survey response rate was low: 54 of 941 invited individuals participated (5.7%). Only 18.6% of PCPs were very familiar or extremely familiar with PrEP, 44% reported at least one PrEP experience, and only 24% had prescribed PrEP in the last 12 months (Table 2). Among those who had prescribed PrEP, 90% had prescribed it to less than 5 patients in the last year. The sample included 31 family physicians, 7 physicians in training, 5 specialists, and 11 nurses (registered nurses or nurse practitioners). Overall, 60% of the PCPs were between 25 and 44 years old; 43% had under 5 years of practice, 10% self-identified as gay, lesbian, queer, or non-binary, and 35% identified as a minority due to religion, race, language, or gender. Among those working in teams or organizations (40/54), 18 (45%) worked for organizations already providing PrEP services; 41% reported colleagues offering PrEP, 27% were unaware of PrEP services at their organization, and 27% reported their organization did not offer PrEP.

**Table 2.** Description of Participants According to Sociodemographic and Practice Characteristics.

Characteristics	N	%
Total		
Familiarity with PrEP		
Not familiar at all	9	15.2
Slightly familiar	22	37.3
Moderately familiar	17	28.8
Very familiar	8	13.5
Extremely familiar	3	5.1
PrEP experience		
Referred	10	18.5
Start a conversation	11	38.9
Prescribed PrEP	13	24.07
Total experience(any of above)	24	44.4
Demographics		
Age		
Less than 25	3	5.4
25-34	22	40.0
35-44	11	20.
45-54	15	27.7
55-64	3	5.4
More than 65	1	1.8
Gender		
Male/cisgender	17	32.7
Female/cisgender	30	58.2
Other	5	9.1
Minority status		
No	36	64.3
Yes (religious, gender, race, language)	20	35.7
Practice characteristics		
Profession		
Family physician	31	56.4
Nurse	10	18.2
Physician in training	7	12.7
Nurse practitioner	1	1.82
Physician specialist	5	9.1
Other	1	1.8
Setting (non-exclusive categories)		
Sexual health clinics	8	14.5
Community settings	2	3.7
Family health teams	11	20.0
Hospital/academic settings	13	23.6
Working with students	7	12.7
Solo practice/physicians in training	14	25.4
Years of practice		
5 years or less	24	43.6
6-10 years	6	10.9
More than 10 years	25	45.5
Size of practice		
Up to 500	21	39.6
500-1000	19	35.8
100-2000	10	18.9
More than 2000	3	5.6
Size population		
More than 100 000	28	50.9
30 000-99 999	15	27.7
1000, 29,999	11	30.0
Less than 1000	1	1.8
Practice with racial minorities		
Less than 10%	33	60
10-20%	8	14.5
More than 20%	5	9.09
Do not know	9	16.4
Practice with gender minorities		
Less than 10%	28	50.9
10-20%	11	20.0
More than 20%	2	3.64
Do not know	14	25.4
Practice with people who use drugs		
Less than 10%	21	38.2
10-20%	14	25.4
More than 20%	5	9.09
Do not know	15	27.7
Organization provide PrEP services		
Yes	18	45.0
No	11	27.5
Do not know	11	27.5
Colleagues provide PrEP		
Yes	17	41.5
No	6	14.6
Do not know	18	43.9

* Abbreviation: PrEP experience: has referred, prescribed, or initiated a conversation on PrEP.

### Description of CFIR Statements and CIBER Analysis

**Figure 1.**
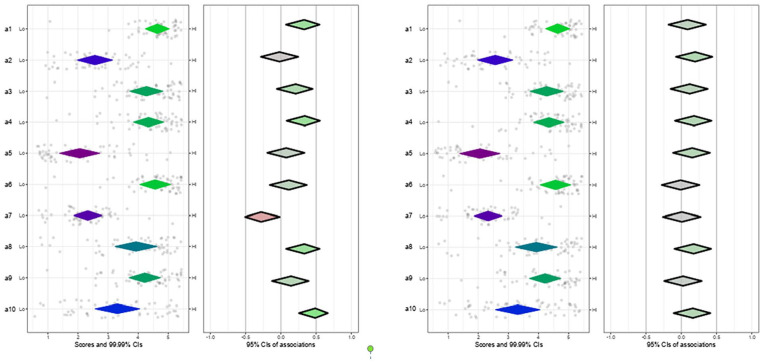
Plot of attitudes toward characteristics of PrEP, Familiarity and Experience with PrEP. The CIBER is a data visualization method that presents different information on a diamond plot to facilitate the selection of the sub-determinants for intervention development. The diamond plot is divided into left-hand panel and right-hand panels with diamonds. One diamond shape in the left-hand panel represents both the means of the sub-determinants (in this study, the CFIR items) and its 99.99% confidence interval, while each diamond in the right-hand panel presents the associations (e.g., correlation) between each of the CFIR items and the outcome variable (in this study the experience with PrEP) with a 95% confidence interval. The dots around the left-hand panel diamonds are all the participants’ item scores.

*PrEP Characteristics*: Participants generally viewed PrEP positively, particularly in terms of its effectiveness and relative advantages (Table S1). Nearly half of the participants thought that PrEP is easy to implement. Those more familiar with PrEP expressed more positive attitudes toward its effectiveness (items a1 and a4), relative advantage (a7), cost-effectiveness compared to HIV treatment (a8), and simplicity (a10) (Figure 1). There were no discernible differences in attitudes toward PrEP between participants with and without prior PrEP experience.

**Figure 2.**
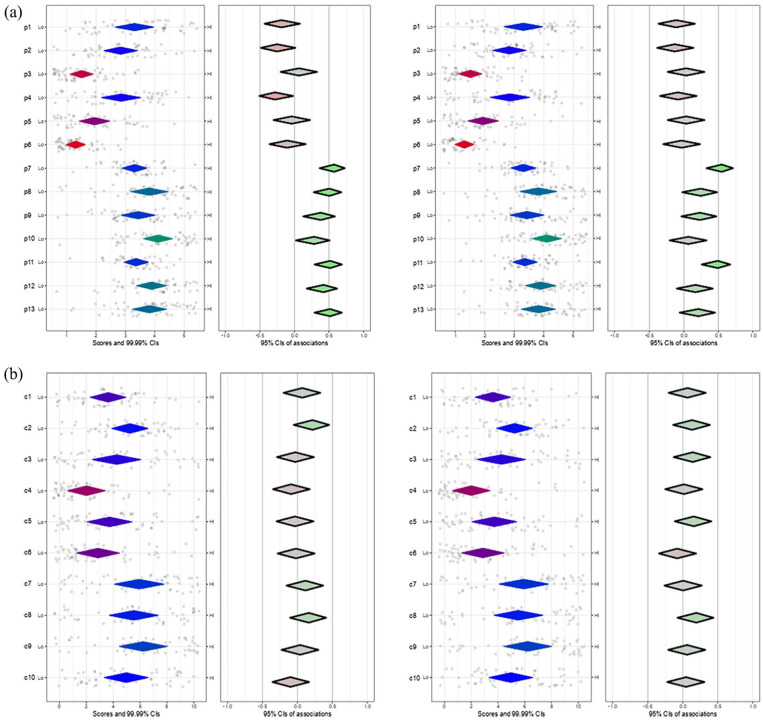
(a) Plot of attitudes toward population needs, Familiarity, and Experience with PrEP and (b) plot of concerns about the use of PrEP, Familiarity, and Experience with PrEP.

*Outer setting*: Participants showed strong positive perceptions regarding PrEP necessity in populations, with statement p10 denoting a perception that “the priority populations want PrEP” having the highest level of agreement (Table S2). Higher familiarity with PrEP correlated with increased agreement on its interest, adaptability, and feasibility among populations (Figure 2a). Participants less familiar with PrEP tended to agree more with the statements “lack of affordability” (p2) and “not a high need” (p4). Experience with PrEP was strongly associated with agreement with the statements “patient wants PrEP” (p7) and “the clinic population wants PrEP” (p11) (Figure 2a). Concerns about PrEP were generally low, particularly for “medication misuse” (c4) and “PrEP-related stigma” (c8), while higher concerns related to access, cost, coverage, and patient monitoring (Table S3). No associations were observed in the distribution of concerns between those with and without PrEP familiarity nor between those with and without PrEP experience (Figure 2b).

**Figure 3.**
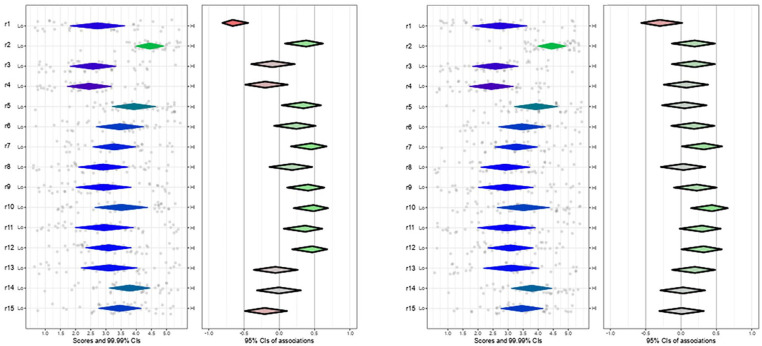
Plot of inner settings domain, familiarity and experience with PrEP.

*Inner setting*: Nearly one-third of participants felt their organizations were not ready to prescribe PrEP (Table S4), 23% identified sexual health clinics as optimal for PrEP delivery, and 21% suggested dedicated facilities. However, 65% agreed that their clinic should offer PrEP. Regarding organization climate, 90% agreed that their colleagues would approve if they prescribed PrEP (r2), 70% think their organization has a preventive focus (r6), but only 22% agree that colleagues would collaborate with PrEP services (r7). Resource limitations were noted by nearly 30% of participants (Table S4). Statements regarding leadership engagement were more neutral or positive (Table S5). Participants familiar with PrEP had more positive perceptions of “approval by colleagues” (r2), “compatibility” (r5), “collaboration by colleagues” (r7), “time to deliver PrEP” (r9), “having resources” (r10) and “having adequate staff” (r12); and less negative perceptions regarding “lack of readiness” (r1) (Figure 3). Participants with PrEP experience had higher positive perceptions of, r7, r10, and r12, and disagreed with r1 when compared to those without PrEP experience (Figure 3).

**Figure 4.**
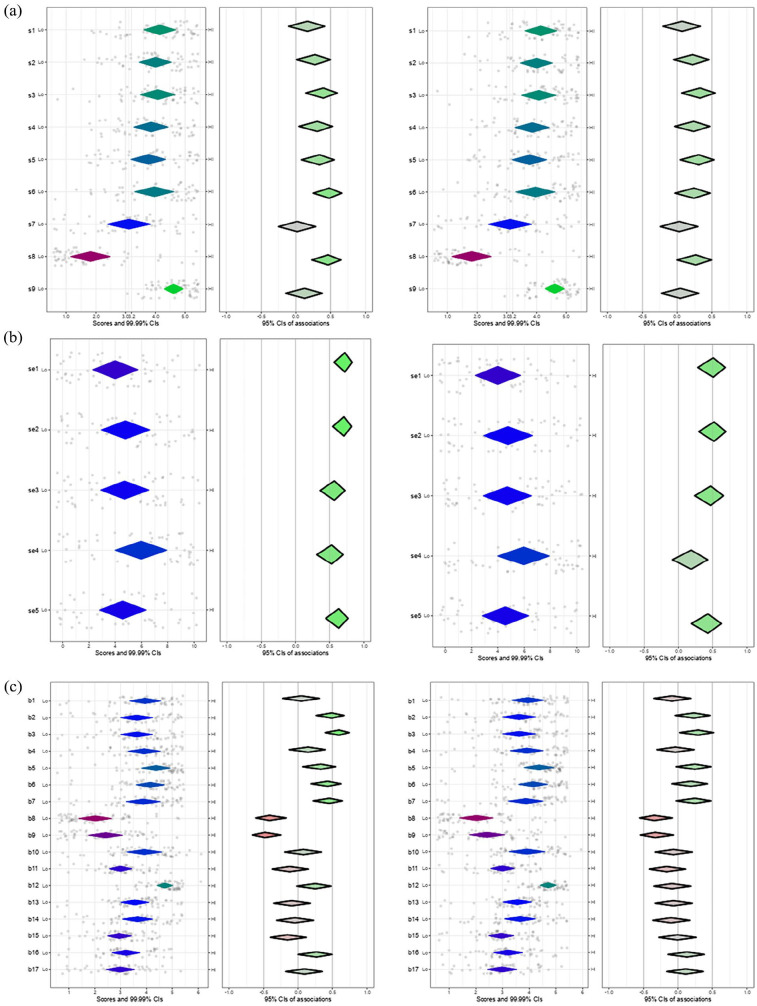
(a) Plot of skills, familiarity, and experience with PrEP, (b) plot of ability or self-efficacy, familiarity and experience with PrEP, and (c) plot of beliefs about consequences, beliefs about compatibility, Familiarity, and Experience with PrEP.

*Individual characteristics*: Participants reported low comfort managing antiretroviral drugs (s7) and disclosing HIV-positive diagnoses (s8) (Table S6). Perceived capability for most PrEP-related activities was below average (score <5) (Table S7). Most PrEP activities were directly related to greater familiarity and experience (Figure 4a and b). Overall, participants had positive beliefs about compatibility (Table S8); the highest relating to the responsibility of PCPs to offer PrEP services (b5). Those more familiar with PrEP agreed more with the statements “good fit” (b2), “compatible with work” (b3), “responsibility to provide PrEP” (b5), “worthiness” (b6), and “should be part” (b7) and more disagreement with “not useful” (b8), and “not a priority” (b9). Participants with PrEP experience disagreed more with b8 and b9 (Figure 4c). Greater positivity about consequences was observed for “help reduce HIV” (b12), with greater variability in item “billing for managing PrEP” (b10) (Table S9). Participants familiar with PrEP tended to agree more with “helping patients” (b12) and “relationship with colleagues” (b16). No differences were found in beliefs about consequences between those with and without PrEP experience (Figure 4c).

### Bivariate and Multivariate Analyses for Demographic and Practice-Related Variables

PrEP familiarity and experience were more common among PCPs who served a higher proportion of gender-minority patients, worked in organizations that provided PrEP, or had colleagues who prescribed PrEP (see Table 3). Additionally, both age and years of practice were positively associated with PrEP experience. Across the entire sample, the optimal model for predicting PrEP familiarity (based on AIC/BIC) included years of practice and having a colleague who prescribed PrEP. Regarding PrEP experience, the best model included “setting” and “provision of PrEP in the organizations (results not shown).

**Table 3.** Bivariate Poisson Regression of Demographic and Practice Related Variables Domains on PrEP Familiarity and Experience.

Variables	% with PreP familiarity	Prevalence ratio (PR) PrEP familiarity	Confidence interval 95%	% with PrEP experience	PR PrEP experience	Confidence interval 95%
Demographics						*
Age		1.10	0.95-1.28		1.32	1.02-1.71
Gender						
Male/cisgender	44.4	Reference	Reference	35.2	Reference	Reference
Female/cisgender	50.0	1.12	0.60-2.01	56.7	1.64	0.78-3.42
Other	60.0	1.35	0.55-3.28	20.0	0.6	0.09-3.96
Minority status						
No	57.1	ref	**	51.4		
Yes (religious, gender, race, language)	35.0	0.61	0.31-1.19	36.8	0.70	0.35-1.39
Practice characteristics						
Profession						
Family physician	51.6	reference	reference	43.3	Reference	reference
Nurse/NP	75.0	1.45	0.85-2.46		1.70	0.90-3.2
Physician in training	40.0	0.77	0.24-2.41	14.3	1.43	0.62-3.29
Physician specialist	28.6	0.55	0.16-1.89	60.0	0.34	0.05-2.22
Other	25.0	0.48	0.08-2.77	0	1.19	0.40-3.48
Setting						
Sexual health clinics	66.7	reference	reference	71.4	reference	reference
Community settings	80.0	1.20	0.63-2.28	100	0.80	0.34-1.83
Family health teams	30.0	0.45	0.15-1.30	54.5	0.80	0.41-1.53
Hospital/academic settings	54.5	0.81	0.39-1.67	38.4	0.36	0.12-1.04
Working with students	50.0	0.75	0.29-1.90	71.4	0.88	0.44-1.78
Solo practice/physicians in training	35.7	0.22	0.30-1.25	15.4	0.19	0.04-0.73
Years of practice						
5 years or less	45.8	reference	reference	29.2	reference	*
6-10 years	66.7	1.45	0.70-2.98	83.3	2.85	1.38-5.90
More than 10 years	48.0	1.04	0.57-1.91	54.5	1.71	0.81-3.62
Size of practice						
Up to 500	42.6	reference	reference	42.1	reference	reference
500-1000	47.4	1.10	0.55-2.20	27.7	0.65	0.25-1.67
100-2000	50.0	1.16	0.52-2.59	70.0	1.75	0.88-3.45
More than 2000	66.7	1.55	0.60-4.01	66.7	1.66	0.62-4.40
Size population						
More than 100 000	53.5	reference	reference	50.0	Reference	reference
30 000-99 999	33.3	0.62	0.27-1.38	42.8	0.83	0.39-1.74
Less than 1000, 29,999	58.3	1.08	0.60-1.97		0.86	0.39-1.89
Practice with racial minorities						
**Less than 10%**	45.4	reference	reference	41.9	reference	reference
10-20%	62.5	1.37	0.71-2.66	50.0	1.23	0.54-2.78
More than 20%	40.0	0.88	0.28-2.77	25.0	0.49	0.07-3.02
Do not know	55.5	1.22	0.60-2.46	66.7	1.64	0.87-3.07
Practice with gender minorities			*			*
Less than 10%	46.4	reference	reference	42.3	reference	reference
10-20%	54.5	1.17	0.59-2.31	54.5	1.38	0.67-2.84
More than 20%	100	2.15	1.44-3.21	100.0	2.54	1.59-4.05
Do not know	42.8	0.92	0.44-1.91	42.8	1.09	0.50-2.34
Practice with people who use drugs**						
Less than 10%	38.1	Reference	Reference	36.8	Reference	Reference
10-20%	57.1	1.50	0.73-3.06	42.8	1.28	0.54-3.04
More than 20%	80.0	2.10	1.03-4.24	80.0	2.40	1.12-5.10
Do not know	46.7	1.22	0.56-2.65	50.0	1.5	0.66-3.36
Organization provide PrEP services			**			*
Yes	72.2	reference	reference	82.3	Reference	reference
No	9.1	0.12	0.01-0.85	40.0	0.44	0.19-1.00
Do not know	63.7	0.88	0.51-1.50	27.3	0.33	0.12-0.90
Colleagues provide PrEP			*			*
Yes	76.5	reference	reference	81.2	Reference	reference
No	0.0	–	–	40.0	0.41	0.12-1.32
Do not know	50.0	0.65	0.38-1.11	38.9	0.48	0.25-0.90
Familiarity with PrEP						*
Not familiar at all				0	—	—
Slightly familiar				31.8	Reference	reference
Moderately familiar				50.0	1.75	0.75-4.06
Very familiar				100	3.49	1.76-6.92
Extremely familiar				100	3.49	1.76-6.92

* Global test *P* value less than .05.

** *P* value <.10, >.05.

### Distribution and Correlations of CFIR Scales

Table 3 presents 8 scales derived by summing the statements within each CFIR domain or subdomain. Significant correlations (Spearman, adjusted by Bonferroni) were observed across these scales. Specifically, the “Characteristics of PrEP” scale showed strong correlations with the “Population Needs” scale (r = 0.66, *P* < .001), “Self-Efficacy” (r = 0.48, *P* = .006), and “Compatibility” (r = 0.79, *P* < .001). The “Population Needs” scale also significantly correlated with “Self-Efficacy” (r = 0.41, *P* = .05) and “Compatibility” (r = 0.57, *P* < .001). Additionally, the “Skills” scale correlated with “Self-Efficacy” (r = 0.51, *P* = .001), and the “Self-Efficacy” scale correlated with “Compatibility” (r = 0.50, *P* = .002).

### Bivariate and Multivariate Analyses for CFIR Variables

Across the entire sample, familiarity and experience with PrEP were significantly associated with 6 of the 8 CFIR scales, with no bivariate relationships found between these outcomes and the “Concerns” or “Beliefs about Consequences” scales (see Table 4). The “Self-Efficacy” and “Attitudes Toward Population Needs” scales provided the most accurate predictions for both PrEP familiarity and experience. In the subsample of individuals working in organizations, the “inner setting” scale was not related to familiarity but provided a good AIC/BIC for PrEP experience (PR = 2.00 95% CI: 0.97-4.11) jointly with “Attitudes Toward Population Needs” (PR = 3.99; 95% CI: 1.29-12.2) and the scale of “Beliefs about consequences” (PR = 0.53; 95% CI: 0.35-0.80).

**Table 4.** Bivariate Poisson Regression of CFIR Related Variables Domains on PrEP Familiarity and Experience.

Variables	Mean, SD and range	Prevalence ratio (PR) familiarity	Confidence interval 95%	PR experience*	Confidence interval 95%
1. Characteristics of PrEP	3.5 (0.5), 1-5	2.25	1.12-4.52	2.01	1.00-4.07
2. Outer settings					
2a.Population needs	3.03 (0.39), 1-3.6	4.15	1.60-10.7	3.7	1.39-9.8
2b. Population concerns	4.4 (1.79); 0.58- 8	1.04	0.89-1.21	1.1	0.92-1.28
3. Inner setting^a^	3.23 (0.45); 2.4-4	1.54	0.78-3.03	1.96	1.02-3.72
4. Individual characteristics					
4a.Skills	3.7(0.77); 1.7-5	1.79	1.28-2.52	1.42	0.95-2.10
4b.Self-efficacy	4.8 (2.8); 0-9.6	1.28	1.16-1.41	1.24	1.10-1.39
4c.Compatibility	3.68 (0.66); 1-4.6	1.97	1.17-3.32	1.38	0.86-2.23
4d. Beliefs about consequences	3.37 (0.49); 2.0-4.35	0.78	0.47-1.29	0.80	0.45-1.41
Familiarity with PrEP				1.77	1.44-2.18

Abbreviation: PrEP experienced, has referred, prescribed, or initiated a conversation on PrEP.

a Sample of 40 PCPs who work for a team clinic or organization.

## Discussion

Regarding our first objective, the level of PrEP familiarity in this sample of PCPs was low, with only 18.6% reporting being very or extremely familiar with PrEP, and 44% having some PrEP experience: initiating PrEP discussions, completing PrEP referrals, or prescribing PrEP. These results align with previous data from Nova Scotia between 2018 and 2019,^27^ where 18% of PCPs had never heard of PrEP and 30% never prescribed it, and with earlier reports (2014) on PCPs’ intent to prescribe PrEP.^37^ These findings underscore that, despite the availability of PrEP guidelines and ongoing national and provincial PrEP initiatives, PrEP adoption remains limited among PCPs in regions outside large metropolitan centers. With regard to the second objective, factors influencing PCPs’ adoption of PrEP, we identified key determinants of PrEP familiarity and experience, including type of practice (e.g., serving special populations), PrEP perceptions (more positive perceptions correlated with greater familiarity and experience), organizational and peer support, clinical skills (such as familiarity with antiretroviral drugs), and beliefs about PrEP’s compatibility and usefulness in their practice.

The sample of PCPs reported predominantly positive perceptions about PrEP and regarded it as a needed intervention, suggesting there is an opportunity to enhance PrEP capacity in their practice settings. We found that PCPs with more PrEP experience viewed PrEP as effective and simple. Familiarity with PrEP is a key determinant of prescription^45^ and correlates with positive perceptions of its effectiveness and simplicity,^22,46,47^ greater self-efficacy and resulting prescription.^48^ Helping PCPs recognize a fast, simple process of PrEP prescription or referral may enhance its use.^49^ In this sample, PCPs with higher familiarity and experience also had more positive views about PrEP need, adaptability, and acceptability among patients, a finding that is consistent with other studies.^27^ However, concerns about the use of PrEP in populations were not related to PrEP familiarity or experience. Concerns about PrEP use, stigma and risk compensation were low in our participants, contrasting with earlier reports^50^ Providers expressed more concern about unequal access, cost, monitoring, and adherence, which remain persistent challenges despite advances in PrEP guidance and policies in Ontario.^8,34^

Our findings highlight that PCPs with greater familiarity and experience with PrEP tend to be concentrated in organizations serving populations likely to seek sexual health services. For example, 70% of sexual health clinics in this study reported PrEP experience, as well as two-thirds of PCPs from organizations serving students, most commonly through patient referrals. Our study supports that PCPs’ familiarity and experience were influenced by aspects of the institution such as perception of leadership support, staff capacity, and resource availability, as many others have found.^51-53^ A supportive work environment where providers feel encouraged to offer PrEP^54^ and where colleagues hold favorable views on PrEP^55^ is crucial for its integration into practice. However, we found that only 11% of the PCPs working in sexual health clinics prescribed PrEP; 17% of the PCPs serving student populations had prescribed PrEP. Additional resources in terms of time, accessible laboratory services, and skilled staff have facilitated the adoption of PrEP in clinical and public health institutions,^52,56,57^ and need to be discussed as strategies to increase PrEP prescription by PCPs working with priority populations.

Importantly, and despite the existent PrEP guidelines and the availability of online resources for PrEP training in Canada, half of the participants reported low comfort in essential PrEP-related tasks, such as disclosing an HIV diagnosis and managing antiretrovirals, aspects that have been previously found to prevent PrEP prescription in other studies.^26^ Skills in testing, counseling, and managing HIV medications were associated with both familiarity and experience with PrEP in our sample as in other studies.^58,59^ Early adopters of PrEP demonstrate a higher level of skills in sexual taking history and sexual minority competence,^59^ findings also related to the experience of PrEP in our sample of PCPs. Proficiency in managing antiretroviral drugs is typically observed in early PrEP adopters and is a crucial skill to gain for PCPs when becoming PrEP prescribers.^60,61^ Perceptions of self-efficacy in PrEP-related activities such as counselling, managing side effects, and identifying eligible PrEP candidates are additional aspects that need further strengthening in PCPs seeking to become PrEP prescribers.^26,62,63^

Lastly, PCPs with greater PrEP familiarity and experience tended to perceive PrEP as compatible with their work, a finding that others have also reported.^41,42,61,64^ Overall, participants in this study viewed sexual health clinics and primary care practices as suitable venues for PrEP services which contrasts to earlier reports where PCPs identified HIV-specialist practices as the best sites for PrEP services.^22^ Regional initiatives, such as nurse-led clinics^15^ and integrative services between 1 sexual health clinic and PCPs^65^ may have contributed to improve the perceptions of feasibility in primary care and public health settings for provision of PrEP in Ontario. One third of our sample was hesitant or disagreeable with their role in PrEP provision, a finding that suggests that emphasis needs to be given to help PCPs recognize their key role in increasing awareness and access to PrEP.

### Limitations and Strengths

This study faced a significant challenge with a low participation rate (<6%), despite efforts to enhance recruitment, which limited the ability of our analyses to identify the factors influencing PrEP adoption among PCPs. This participation rate in this study falls within the range of 2%-21% in previous surveys of Canadian PCPs.^66^ Recent surveys indicate deteriorating mental health and professional fulfillment among PCPs, potentially reducing their inclination to participate in research. Due to the small sample size, we could not accurately estimate PrEP prescribing prevalence or identify barriers preventing PCPs familiar with PrEP from prescribing it. Thus, findings may overrepresent those with a vested interest in PrEP or familiarity with HIV management, limiting generalizability. Nonetheless, the insights gathered reflect local attitudes and needs and are consistent with broader research.^22,40,47,52^ The evidence presented here will add to that from this study’s qualitative research (reported elsewhere) and will be interpreted alongside those results to inform interventions intended to increase PCPs’ PrEP adoption.

### Implications

Our findings indicate a need for capacity building on HIV PrEP to promote higher adoption and expansion of PrEP services to smaller cities, suburban, and rural settings. Many PCPs currently referring patients for PrEP could be empowered to transition to PrEP prescribing. Implementation strategies targeting individual and organizational barriers and facilitators could include (i) teaching skills conducive to PrEP adoption, (ii) enhancing PCPs’ understanding of local populations, risk factors, and PrEP barriers, and (iii) addressing concerns related to adherence, coverage, and access to PrEP. Online training modules, coupled with expert peer support (e.g., phone advice, case discussions) medication funding tools, and medical directive templates, show promise in promoting PCPs’ broader PrEP adoption.^67^ Educational institutions with large student populations could significantly expand the pool of PrEP prescribers by integrating comprehensive sexual health programs that include HIV and STI prevention, including PrEP. Effective training and education efforts must be supported by increased resources for delivering PrEP services, utilizing innovative models such as task-shifting and involving key populations.^32,68^ Lastly, structural approaches are essential to enhance PrEP access in underserved communities.^25,28^ Those may include changing physician remuneration, allocating funding for public health and sexual health programs, free-of-charge PrEP on sites providing STI services, addressing HIV and PrEP stigma,^69^ and levering communities and collaboration.^28,32^

## Conclusion

Primary care providers in Ontario are crucial for expanding PrEP implementation but require reinforcement of education to acquire new skills for PrEP delivery. Enhancing organizational capacity and culture for PrEP should involve innovative delivery methods, training of PrEP champions, and collaboration with local leaders and communities.

## Supplemental Material

sj-docx-1-jpc-10.1177_21501319251315566 – Supplemental material for Determinants of Familiarity and Experience with HIV Pre-Exposure Prophylaxis in Primary Care Providers in Ontario, Canada Supplemental material, sj-docx-1-jpc-10.1177_21501319251315566 for Determinants of Familiarity and Experience with HIV Pre-Exposure Prophylaxis in Primary Care Providers in Ontario, Canada by Jorge Martinez-Cajas, Beatriz Alvarado, Carmela Rapino, Emma Nagy, T. Hugh Guan, Nicholas Cofie, Nancy Dalgarno, Pilar Camargo and Bradley Stoner in Journal of Primary Care & Community Health

sj-docx-2-jpc-10.1177_21501319251315566 – Supplemental material for Determinants of Familiarity and Experience with HIV Pre-Exposure Prophylaxis in Primary Care Providers in Ontario, Canada Supplemental material, sj-docx-2-jpc-10.1177_21501319251315566 for Determinants of Familiarity and Experience with HIV Pre-Exposure Prophylaxis in Primary Care Providers in Ontario, Canada by Jorge Martinez-Cajas, Beatriz Alvarado, Carmela Rapino, Emma Nagy, T. Hugh Guan, Nicholas Cofie, Nancy Dalgarno, Pilar Camargo and Bradley Stoner in Journal of Primary Care & Community Health
