# Supplementary material for: Determinants of Familiarity and Experience with HIV Pre-Exposure Prophylaxis in Primary Care Providers in Ontario, Canada
Source: J Prim Care Community Health. 2025 Jan 23;16:21501319251315566. doi: 10.1177/21501319251315566 (PMC11755537; doi:10.1177/21501319251315566)
Supplement: sj-docx-1-jpc-10.1177_21501319251315566 – Supplemental material for Determinants of Familiarity and Experience with HIV Pre-Exposure Prophylaxis in Primary Care Providers in Ontario, Canada [file sj-docx-1-jpc-10.1177_21501319251315566.docx]

PrEP PCP SEO

End of Block: consent form

Start of Block: screening knowledge of prep

Q2.1 Since this study focus on primary care practitioners in Ontario, we need to know the city or town where you practice:

________________________________________________________________

Q2.2 Before this survey....

- I had never heard about PrEP (HIV Pre-exposure prophylaxis) (5)
- I had heard about PrEP (2)

Q2.3 Please select the statement that best applies to you regarding your familiarity with HIV pre-exposure prophylaxis (PrEP)

- Not familiar at all (14)
- Slightly familiar (15)
- Moderately familiar (16)
- Very familiar (17)
- Extremely familiar (18)

| Page Break |  |
| --- | --- |

End of Block: screening knowledge of prep

Start of Block: awareness of ontario website

Q3.1 The following questions refer to your previous educational experiences in gaining knowledge of PrEP.

| Page Break |  |
| --- | --- |

Q3.2 Various guidelines are available to help practitioners with management of PrEP patients. From the list below, mark all that you have reviewed.

- I have not read any guidelines (6)
- American/ CDC guidelines (1)
- Canadian guidelines (2)
- British Columbia Guidelines (3)
- WHO guidelines (4)
- Guidelines presented in clinical practice tools (e.g UptoDate) (7)
- Other guidelines or resources, which ones? (5) __________________________________________________

| Page Break |  |
| --- | --- |

Q3.3 Were you aware of the ontarioprep.ca website and its PrEP educational resources?

- Yes, I have used them (2)
- Yes, but I have not used them (3)
- No, I was not aware they were available (1)

Skip To: End of Block If Q3.3 = No, I was not aware they were available

Skip To: End of Block If Q3.3 = Yes, but I have not used them

| Page Break |  |
| --- | --- |

Q3.4 To what extent do you agree or disagree that training through OntarioPrEP.ca enabled you to

|  |  |  |  |  |  |  |
| --- | --- | --- | --- | --- | --- | --- |
| Start a conversation about PrEP with a patient (1) | - Strongly Disagree (1) | - Disagree (2) | - Slightly Disagree (3) | - Slightly Agree (4) | - Agree (5) | - Strongly Agree (6) |
| Start PrEP in a patient (2) | - Strongly Disagree (1) | - Disagree (2) | - Slightly Disagree (3) | - Slightly Agree (4) | - Agree (5) | - Strongly Agree (6) |
| Counselling PrEP candidates (3) | - Strongly Disagree (1) | - Disagree (2) | - Slightly Disagree (3) | - Slightly Agree (4) | - Agree (5) | - Strongly Agree (6) |
| Monitoring patients on PrEP (4) | - Strongly Disagree (1) | - Disagree (2) | - Slightly Disagree (3) | - Slightly Agree (4) | - Agree (5) | - Strongly Agree (6) |

| Page Break |  |
| --- | --- |

Display This Question:

If Q3.3 = Yes, but I have not used them

Q3.5 What prevented you from using the ontarioprep.ca educational resources?

________________________________________________________________

________________________________________________________________

________________________________________________________________

________________________________________________________________

________________________________________________________________

| Page Break |  |
| --- | --- |

Q3.6 What aspects of  ontarioprep.ca would you change that could make it easier for you to start offering PrEP services? (answer "none" if appropriate)

________________________________________________________________

________________________________________________________________

________________________________________________________________

________________________________________________________________

________________________________________________________________

End of Block: awareness of ontario website

Start of Block: PrEP cascade

Q4.1 The following questions will help us to determine your experience with PrEP care.
 
***In the last 12 months:***

|  | Yes (1) | No (2) |
| --- | --- | --- |
| Have any of your patients asked you about PrEP? (4) |  |  |
| Have you initiated a conversation on PrEP with a patient? (3) |  |  |
| Have you started a patient on PrEP? (1) |  |  |
| Have you referred a patient to a PrEP provider? (2) |  |  |

End of Block: PrEP cascade

Start of Block: introduction to PREP' only for those who does not know

Q5.1   What is HIV PrEP (HIV Pre-exposure prophylaxis)?   PrEP involves the administration of specific antiretroviral medications to persons who are not HIV infected with the goal of preventing HIV acquisition. Typically, in persons who take PrEP are at high and ongoing risk of acquiring HIV, start these medications before exposure to HIV and continue them for some time after potential HIV exposures.   Most often, PrEP medications are taken orally, but parenteral and intravaginal options have also become available. The efficacy of oral and parenteral PrEP medications has been demonstrated in several randomized, double-blind clinical trials. Oral PrEP has been recommended by authoritative guidelines in Canada and around the world.   For instance, daily oral tenofovir disoproxilfumarate/emtricitabine can reduce the risk of HIV acquisition in over 90% in individuals with high adherence.    PrEP differs from post-exposure prophylaxis (PEP) which refers to taking antiretroviral medications on a daily basis for 28 days following a possible HIV exposure.

End of Block: introduction to PREP' only for those who does not know

Start of Block: intentions

| Page Break |  |
| --- | --- |

Q6.1 In the next 12 months, how likely is it that you will manage PrEP patients in the clinic where you work?

- Extremely unlikely (21)
- Somewhat unlikely (22)
- Neither likely nor unlikely (23)
- Somewhat likely (24)
- Extremely likely (25)

Carry Forward All Choices - Displayed & Hidden from "Q10.2"

| 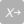 |
| --- |

Q6.2 In the next 12 months, how likely is it that you take over the management of PrEP for patients started by another practitioner?

- Extremely unlikely (1)
- Somewhat unlikely (2)
- Neither likely nor unlikely (3)
- Somewhat likely (4)
- Extremely likely (5)

| Page Break |  |
| --- | --- |

End of Block: intentions

Start of Block: Referred patients for prep

| Page Break |  |
| --- | --- |

Q7.1 To whom have you referred a patient for PrEP (e.g., to a PrEP provider or HIV clinic)?
***Mark all that apply***

- an infectious disease doctor (1)
- a known PrEP provider (2)
- a PrEP provider identified through internet or another search (e.g., at the Ontario PrEP website) (4)

| 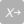 |
| --- |

Q7.2 How many of your patients have you referred for PrEP ***in the last year***?

- Less than 5 (1)
- Between 5 and 10 (2)
- More than 10 (3)

| Page Break |  |
| --- | --- |

Q7.3 Has any of the patients that you referred to a PrEP provider come back to your clinic to continue PrEP care?

- Yes (1)
- No (2)

|  |  |
| --- | --- |

End of Block: Referred patients for prep

Start of Block: initiated a conversation

| Page Break |  |
| --- | --- |

| 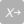 |
| --- |

Q8.1 How many times in the last year did you initiate a conversation about PrEP?

- At least once a week (1)
- One to three times a month (2)
- Four to ten times per year (3)
- Three or fewer times per year (4)

Q8.2  With whom did you initiate a conversation about PrEP? ***(mark all that apply)***

- Gay/bisexual men (1)
- Transgender people (6)
- People who are sex workers (5)
- People who inject drugs (7)
- People who are incarcerated (8)
- Heterosexual men (9)
- Heterosexual women (12)
- HIV serodiscordant couples (10)
- Other: (11) __________________________________________________

| Page Break |  |
| --- | --- |

End of Block: initiated a conversation

Start of Block: asked about prep

| 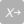 |
| --- |

Q9.1 How many times ***in the last year*** were you asked about PrEP?

- At least once a week (1)
- One to three times a month (2)
- Four to ten times per year (3)
- Three or fewer times per year (4)

| Page Break |  |
| --- | --- |

End of Block: asked about prep

Start of Block: Prescribed prep

| Page Break |  |
| --- | --- |

| 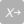 |
| --- |

Q10.1 To how many patients did you prescribe PrEP in the last year?

- Less than 5 (1)
- Between 5 and 10 (2)
- More than 10 (3)

| Page Break |  |
| --- | --- |

Q10.2 In the next 12 months, how likely is it that you will continue to prescribe PrEP?

- Extremely unlikely (19)
- Somewhat unlikely (20)
- Neither likely nor unlikely (21)
- Somewhat likely (22)
- Extremely likely (23)

End of Block: Prescribed prep

Start of Block: Barriers for prep services

Q11.1 Have you encountered any of the following obstacles to providing PrEP care?
***Check all that apply***

- Lack of clinical protocols or guidelines for providing PrEP care (1)
- Lack of clinical protocols to gain understanding of the provision of PrEP care (10)
- Lack of access to training/education regarding PrEP (2)
- Difficulties with monitoring (3)
- Staff/time constrains to offer risk reduction or adherence counseling (4)
- Lack of insurance coverage (5)
- On-site support (i.e. risk reduction, counselors, social workers) (6)
- Practice or institutional willingness to implement clinical protocols (7)
- Practice environment supportive of PrEP provision (8)
- Other, which ones? (9) __________________________________________________

End of Block: Barriers for prep services

Start of Block: CFIR-intervention characteristics

Q12.1 How much do you agree or disagree with the following statements?

|  |  |  |  |  |  |
| --- | --- | --- | --- | --- | --- |
| PrEP prevents the transmission of HIV (1) | - Strongly disagree (1) | - Slightly disagree (2) | - Neither agree nor disagree (3) | - Slightly agree (4) | - Strongly agree (5) |
| PrEP has the potential to do more harm than good if not carefully implemented (5) | - Strongly disagree (1) | - Slightly disagree (2) | - Neither agree nor disagree (3) | - Slightly agree (4) | - Strongly agree (5) |
| The use of PrEP is well supported by evidence (6) | - Strongly disagree (1) | - Slightly disagree (2) | - Neither agree nor disagree (3) | - Slightly agree (4) | - Strongly agree (5) |
| PrEP is an effective prevention method and should be widely used as soon as possible (8) | - Strongly disagree (1) | - Slightly disagree (2) | - Neither agree nor disagree (3) | - Slightly agree (4) | - Strongly agree (5) |
| PrEP can lead to the medicalization of HIV prevention and take resources that can be better used somewhere else (11) | - Strongly disagree (1) | - Slightly disagree (2) | - Neither agree nor disagree (3) | - Slightly agree (4) | - Strongly agree (5) |
| I think that PrEP is a good addition to improve the sexual health of populations at risk (27) | - Strongly disagree (1) | - Slightly disagree (2) | - Neither agree nor disagree (3) | - Slightly agree (4) | - Strongly agree (5) |

| Page Break |  |
| --- | --- |

Q12.2 How much do you agree or disagree with the following statements?

|  |  |  |  |  |  |
| --- | --- | --- | --- | --- | --- |
| I think there are better alternatives than PrEP for HIV prevention (28) | - Strongly disagree (1) | - Slightly disagree (2) | - Neither agree nor disagree (3) | - Slightly agree (4) | - Strongly agree (5) |
| I think PrEP is less costly than HIV treatment (29) | - Strongly disagree (1) | - Slightly disagree (2) | - Neither agree nor disagree (3) | - Slightly agree (4) | - Strongly agree (5) |
| I think PrEP would be a good addition to condom use programs (30) | - Strongly disagree (1) | - Slightly disagree (2) | - Neither agree nor disagree (3) | - Slightly agree (4) | - Strongly agree (5) |
| PrEP services will be easy to implement in the clinic where I practice (31) | - Strongly disagree (1) | - Slightly disagree (2) | - Neither agree nor disagree (3) | - Slightly agree (4) | - Strongly agree (5) |

| Page Break |  |
| --- | --- |

Q12.3 As a result of the use of PrEP in populations at risk, how concerned are you about? ***0= Not at all concerned***
***10= Very concerned***

|  | 0 | 1 | 2 | 3 | 4 | 5 | 6 | 7 | 8 | 9 | 10 |
| --- | --- | --- | --- | --- | --- | --- | --- | --- | --- | --- | --- |

| Developing resistance to HIV antiviral medications () | 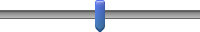 |
| --- | --- |
| Adherence to PrEP medications () | 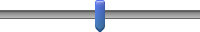 |
| Lower use of condoms () | 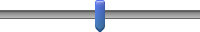 |
| Misuse of medications, e.g selling medications () | 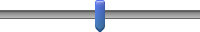 |
| Increase in sexual transmitted diseases other than HIV () | 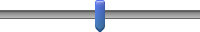 |
| PrEP related stigma () | 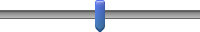 |
| Unequal access to PrEP in some vulnerable population groups () | 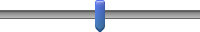 |
| Cost of PrEP medications () | 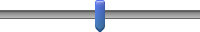 |
| Coverage for PrEP medications () | 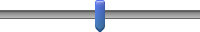 |
| Difficulties in monitoring patients who take PrEP () | 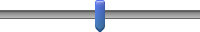 |

End of Block: CFIR-intervention characteristics

Start of Block: CFIR-Population Needs

| 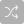 |
| --- |

Q13.1 How much do you agree or disagree with the following statements?

|  |  |  |  |  |  |
| --- | --- | --- | --- | --- | --- |
| I see very few people who may be interested in or potentially benefit from PrEP (1) | - Strongly disagree (1) | - Somewhat disagree (2) | - Neither agree nor disagree (3) | - Somewhat agree (4) | - Strongly agree (5) |
| I do not think that the population I see in my clinic can afford PrEP (5) | - Strongly disagree (1) | - Somewhat disagree (2) | - Neither agree nor disagree (3) | - Somewhat agree (4) | - Strongly agree (5) |
| If PrEP were implemented in the clinic where I practice, this would bother some of my patients or the local public (7) | - Strongly disagree (1) | - Somewhat disagree (2) | - Neither agree nor disagree (3) | - Somewhat agree (4) | - Strongly agree (5) |
| PrEP is not a high need in the area where I practice (9) | - Strongly disagree (1) | - Somewhat disagree (2) | - Neither agree nor disagree (3) | - Somewhat agree (4) | - Strongly agree (5) |
| I do not believe that the population at risk of HIV is interested in using PrEP (13) | - Strongly disagree (1) | - Somewhat disagree (2) | - Neither agree nor disagree (3) | - Somewhat agree (4) | - Strongly agree (5) |
| I believe it is unethical to prescribe antiretrovirals to HIV negative people (16) | - Strongly disagree (1) | - Somewhat disagree (2) | - Neither agree nor disagree (3) | - Somewhat agree (4) | - Strongly agree (5) |

| Page Break |  |
| --- | --- |

| 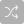 |
| --- |

Q13.2 How much do you agree or disagree with the following statements?

|  |  |  |  |  |  |
| --- | --- | --- | --- | --- | --- |
| PrEP is something that my patients want (6) | - Strongly disagree (1) | - Somewhat disagree (2) | - Neither agree nor disagree (3) | - Somewhat agree (4) | - Strongly agree (5) |
| It is possible to set up PrEP in my practice (10) | - Strongly disagree (1) | - Somewhat disagree (2) | - Neither agree nor disagree (3) | - Somewhat agree (4) | - Strongly agree (5) |
| There is strong buy-in from community organizations for PrEP (12) | - Strongly disagree (1) | - Somewhat disagree (2) | - Neither agree nor disagree (3) | - Somewhat agree (4) | - Strongly agree (5) |
| PrEP is something people at risk of HIV want (14) | - Strongly disagree (1) | - Somewhat disagree (2) | - Neither agree nor disagree (3) | - Somewhat agree (4) | - Strongly agree (5) |
| PrEP is something that people who receive care in my clinic want (15) | - Strongly disagree (1) | - Somewhat disagree (2) | - Neither agree nor disagree (3) | - Somewhat agree (4) | - Strongly agree (5) |
| It is possible to adapt PrEP protocols to the needs of populations at risk of HIV (17) | - Strongly disagree (1) | - Somewhat disagree (2) | - Neither agree nor disagree (3) | - Somewhat agree (4) | - Strongly agree (5) |
| It is possible to set up PrEP in the clinic where I practice (18) | - Strongly disagree (1) | - Somewhat disagree (2) | - Neither agree nor disagree (3) | - Somewhat agree (4) | - Strongly agree (5) |

| Page Break |  |
| --- | --- |

End of Block: CFIR-Population Needs

Start of Block: profesional role/compatibility

| 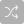 |
| --- |

Q14.1 How much do you agree or disagree with the following statements?

|  |  |  |  |  |  |
| --- | --- | --- | --- | --- | --- |
| I see my values reflected in the implementation of PrEP (11) | - Strongly disagree (1) | - Somewhat disagree (2) | - Neither agree nor disagree (3) | - Somewhat agree (4) | - Strongly agree (5) |
| PrEP will be a very good fit in my clinic (13) | - Strongly disagree (1) | - Somewhat disagree (2) | - Neither agree nor disagree (3) | - Somewhat agree (4) | - Strongly agree (5) |
| Managing PrEP will be compatible with my work in the clinic (14) | - Strongly disagree (1) | - Somewhat disagree (2) | - Neither agree nor disagree (3) | - Somewhat agree (4) | - Strongly agree (5) |
| I see my personal values reflected in the implementation of PrEP (15) | - Strongly disagree (1) | - Somewhat disagree (2) | - Neither agree nor disagree (3) | - Somewhat agree (4) | - Strongly agree (5) |
| It is my responsibility as a primary care provider to offer PrEP services to patients who may need it (16) | - Strongly disagree (1) | - Somewhat disagree (2) | - Neither agree nor disagree (3) | - Somewhat agree (4) | - Strongly agree (5) |
| I think that managing PrEP patients will be worth it (17) | - Strongly disagree (1) | - Somewhat disagree (2) | - Neither agree nor disagree (3) | - Somewhat agree (4) | - Strongly agree (5) |
| Prescribing and/or managing PrEP should be part of the work I do in the clinic (19) | - Strongly disagree (1) | - Somewhat disagree (2) | - Neither agree nor disagree (3) | - Somewhat agree (4) | - Strongly agree (5) |

| Page Break |  |
| --- | --- |

| 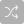 |
| --- |

Q14.2 How much do you agree or disagree with the following statements?

|  |  |  |  |  |  |
| --- | --- | --- | --- | --- | --- |
| For me, managing PrEP will not be useful at all (8) | - Strongly disagree (1) | - Somewhat disagree (2) | - Neither agree nor disagree (3) | - Somewhat agree (4) | - Strongly agree (5) |
| Managing PrEP is not a priority for me (9) | - Strongly disagree (1) | - Somewhat disagree (2) | - Neither agree nor disagree (3) | - Somewhat agree (4) | - Strongly agree (5) |

| Page Break |  |
| --- | --- |

| 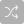 |
| --- |

Q14.3 How much you agree or disagree with the following aspects of PrEP?

|  | Strongly disagree (23) | Somewhat disagree (24) | Neither agree nor disagree (25) | Somewhat agree (26) | Strongly agree (27) | Not applicable (28) |
| --- | --- | --- | --- | --- | --- | --- |
| I can bill for managing PrEP patients (2) |  |  |  |  |  |  |
| If I manage PrEP patients, I can obtain recognition from my colleagues (3) |  |  |  |  |  |  |
| If I manage PrEP patients, I will help my patients to reduce their risk of HIV (4) |  |  |  |  |  |  |
| If I manage PrEP patients, I will get recognition from my patients (5) |  |  |  |  |  |  |
| If I manage PrEP patients, this will strengthen collaboration with other professionals (6) |  |  |  |  |  |  |
| If I manage PrEP patients, I will get recognition in my organization (7) |  |  |  |  |  |  |
| Most people who are important to me think that I should manage PrEP patients (8) |  |  |  |  |  |  |
| By managing PrEP, I would receive recognition from professionals that are important to me (9) |  |  |  |  |  |  |

End of Block: profesional role/compatibility

Start of Block: self efficacy

Q15.1 On a scale from 1 to 10, how confident are you that you are ABLE to perform the following tasks?

|  | Not at all confident |  |  |  | Totally confident | Not Applicable |
| --- | --- | --- | --- | --- | --- | --- |

|  | 0 | 1 | 2 | 3 | 4 | 5 | 6 | 7 | 8 | 9 | 10 |
| --- | --- | --- | --- | --- | --- | --- | --- | --- | --- | --- | --- |

| Offering complete PrEP care () | 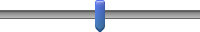 |
| --- | --- |
| Counseling patients about PrEP () | 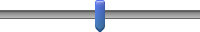 |
| Using the tools to identify patients eligible for PrEP () | 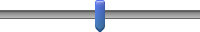 |
| Offering PrEP care if supported with a clear protocol and prescribing tools () | 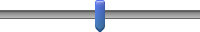 |
| Managing effects of medications used for PrEP () | 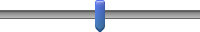 |

16.1 How would you describe your comfort level  with the following clinical practices?

|  | Completely comfortable (1) | Very comfortable (2) | Somewhat comfortable (3) | Slightly comfortable (4) | Not at all comfortable (5) | Not applicable (93) |
| --- | --- | --- | --- | --- | --- | --- |
| Inquiring about sexual orientation (1) |  |  |  |  |  |  |
| Discussing sexual habits with your patients (4) |  |  |  |  |  |  |
| HIV screening (5) |  |  |  |  |  |  |
| Screening for high-risk sexual habits (6) |  |  |  |  |  |  |
| Providing risk reduction counseling (7) |  |  |  |  |  |  |
| Ordering a diagnostic test for acute HIV (8) |  |  |  |  |  |  |
| Revealing an HIV diagnosis to your patient(s) (24) |  |  |  |  |  |  |
| Managing HIV medications (25) |  |  |  |  |  |  |
| Ordering tests for sexually transmitted infections (Chlamydia, syphilis, gonorrhea) (26) |  |  |  |  |  |  |

| Page Break |  |
| --- | --- |

End of Block: Skills, comfort

Start of Block: PEP

| Page Break |  |
| --- | --- |

Q17.1 In the last 12 months did you prescribe or refer patients for HIV post-exposure prophylaxis (PEP)?

- Yes (1)
- No (7)
- I am not allowed to prescribe PEP (6)
- I have referred PEP patients to someone else (e.g. emergency department) (8)

Skip To: End of Block If Q17.1 = No

Skip To: End of Block If Q17.1 = I am not allowed to prescribe PEP

Skip To: End of Block If Q17.1 = I have referred PEP patients to someone else (e.g. emergency department)

| Page Break |  |
| --- | --- |

| 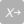 |
| --- |

Q17.2 To how many people did you prescribe PEP in the last year?

- Less than 5 (1)
- Between 5 and 10 (2)
- More than 10 (3)

| Page Break |  |
| --- | --- |

End of Block: PEP

Start of Block: Practice

Q18.1 What is your profession/specialty?

- Family physician (1)
- Nurse practitioner (4)
- Registered Nurse (14)
- Physician/Specialist, please specify (15) __________________________________________________
- Family physician in training (16)
- Other, please specify (6) __________________________________________________

Q18.2 How many hours per week do you do clinical work?

- HOURS per week (1) __________________________________________________

Q18.3 For how many years have you been in active practice ?

- 0-5 years (4)
- 6-10 years (5)
- More than 10 years (6)

| Page Break |  |
| --- | --- |

Q18.4 How would you classify the area or region where you practice?

- Comunity with more than 100,000 people (1)
- Community with 30,000 to 99,999 people (2)
- Community with 1,000 to 29,999 people (3)
- Community with less than 1,000 people (4)

Q18.5  How many patients do you follow in the ambulatory clinic where you practice?

- Up to 500 (1)
- From 500 to 1000 (2)
- From 1000-2000 (3)
- More than 2000 (4)

Q18.6 Of the patients you currently provide care for at your clinic/office, approximately how many are from a racial minority population?

- Less than 10% of my patients (1)
- 10% to 20% of my patients (15)
- More than 20% of my patients (16)
- I do not know (17)

Carry Forward All Choices - Displayed & Hidden from "Q18.6"

| 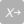 |
| --- |

Q18.7 Of the patients you currently provide care for at your clinic/office, approximately how many are from a sexual/gender minority population, eg. transgender, gay, bisexual, lesbian?

- Less than 10% of my patients (1)
- 10% to 20% of my patients (2)
- More than 20% of my patients (3)
- I do not know (4)

Carry Forward All Choices - Displayed & Hidden from "Q18.6"

| 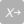 |
| --- |

Q18.8 Of the patients you currently provide care for at your clinic/office, approximately how many are people who use drugs?

- Less than 10% of my patients (1)
- 10% to 20% of my patients (2)
- More than 20% of my patients (3)
- I do not know (4)

End of Block: Practice

Start of Block: Inner settings

Q19.1 The following questions are regarding your perception of the readiness of the clinic where you work to implement PrEP services. If you work exclusively in SOLO practice and you are not affiliated to any organization or clinic, please click the first option below; if not please click the second option.  If you are a physician in training please select the third option.

- I work exclusively in SOLO practice (4)
- I do not work exclusively in SOLO practice (7)
- I am physician in training (8)

Skip To: End of Block If Q19.1 = I work exclusively in SOLO practice

Skip To: End of Block If Q19.1 = I am physician in training

| Page Break |  |
| --- | --- |

Q19.2 Please identify all settings that are major or important parts of your clinical practice.   ***Please mark the three most relevant parts in your practice.***

- Solo practice (15)
- Shared practice (13)
- Sexual health clinic (11)
- Student health service at a university/college (10)
- Clinic dedicated to LGTBIQ2+ health needs (9)
- Clinic at an academic medical center (1)
- Clinic at a public hospital (4)
- Clinic for the armed forces (5)
- Clinic at substance abuse treatment center in the community (6)
- Community health centre (7)
- Inpatient/hospital setting (8)
- Other (14) __________________________________________________

| Page Break |  |
| --- | --- |

Q19.3 Is your clinic or organization currently providing PrEP services?

- Yes (1)
- No (2)
- I do not know (3)

| Page Break |  |
| --- | --- |

Q19.4 In the next 12 months, how likely is it that your clinic will implement protocols or policies support the delivery of PrEP services?

- Extremely unlikely (14)
- Somewhat unlikely (15)
- Neither likely nor unlikely (16)
- Somewhat likely (17)
- Extremely likely (18)

| Page Break |  |
| --- | --- |

Q19.5 Are any of your colleagues in the clinic providing PrEP services?

- Yes (1)
- No (2)
- I do not know (3)

| Page Break |  |
| --- | --- |

Q19.6 In the next 12 months, how likely is it that your colleagues will start to provide PrEP services?

- Extremely unlikely (14)
- Somewhat unlikely (15)
- Neither likely nor unlikely (16)
- Somewhat likely (17)
- Extremely likely (18)

| Page Break |  |
| --- | --- |

Q19.7 In the next 12 months, how likely is it that you will advocate for PrEP in your clinical setting or organization?

- Extremely unlikely (21)
- Somewhat unlikely (22)
- Neither likely nor unlikely (23)
- Somewhat likely (24)
- Extremely likely (25)

| Page Break |  |
| --- | --- |

Q19.8 How much do you agree or disagree with the following statements?

|  |  |  |  |  |  |
| --- | --- | --- | --- | --- | --- |
| PrEP is safe, but we are not ready to prescribe it in the clinic where I work (4) | - Strongly disagree (1) | - Somewhat disagree (2) | - Neither agree nor disagree (3) | - Somewhat agree (4) | - Strongly agree (5) |
| Many colleagues around me would approve the use of PrEP (29) | - Strongly disagree (1) | - Somewhat disagree (2) | - Neither agree nor disagree (3) | - Somewhat agree (4) | - Strongly agree (5) |
| It is more suitable to provide PrEP in sexual health/HIV clinics than in general primary care clinics (30) | - Strongly disagree (1) | - Somewhat disagree (2) | - Neither agree nor disagree (3) | - Somewhat agree (4) | - Strongly agree (5) |
| It is more suitable to provide PrEP in PrEP-dedicated clinics than in general primary care clinics (32) | - Strongly disagree (1) | - Somewhat disagree (2) | - Neither agree nor disagree (3) | - Somewhat agree (4) | - Strongly agree (5) |
| I think that the prescription of PrEP should be part of the work in the clinic (33) | - Strongly disagree (1) | - Somewhat disagree (2) | - Neither agree nor disagree (3) | - Somewhat agree (4) | - Strongly agree (5) |

| Page Break |  |
| --- | --- |

| 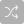 |
| --- |

Q19.9 How much you agree or disagree with the following aspects of PrEP?

|  |  |  |  |  |  |
| --- | --- | --- | --- | --- | --- |
| My organization/clinic is sufficiently oriented towards prevention (3) | - Strongly disagree (1) | - Somewhat disagree (2) | - Neither agree nor disagree (3) | - Somewhat agree (4) | - Strongly agree (5) |
| In the organization/clinic where I work, professionals can collaborate to offer PrEP (5) | - Strongly disagree (1) | - Somewhat disagree (2) | - Neither agree nor disagree (3) | - Somewhat agree (4) | - Strongly agree (5) |
| In the organization/clinic where I work, we see a good number of potential PrEP candidates (7) | - Strongly disagree (1) | - Somewhat disagree (2) | - Neither agree nor disagree (3) | - Somewhat agree (4) | - Strongly agree (5) |
| In the organization/clinic where I work, there is enough time to deliver PrEP services (10) | - Strongly disagree (1) | - Somewhat disagree (2) | - Neither agree nor disagree (3) | - Somewhat agree (4) | - Strongly agree (5) |
| In the organization/clinic where I work, there are enough resources to deliver PrEP (13) | - Strongly disagree (1) | - Somewhat disagree (2) | - Neither agree nor disagree (3) | - Somewhat agree (4) | - Strongly agree (5) |
| My clinic has the necessary resources and support to set up PrEP services (15) | - Strongly disagree (1) | - Somewhat disagree (2) | - Neither agree nor disagree (3) | - Somewhat agree (4) | - Strongly agree (5) |
| My clinic has the necessary staffing to support the implementation of PrEP (17) | - Strongly disagree (1) | - Somewhat disagree (2) | - Neither agree nor disagree (3) | - Somewhat agree (4) | - Strongly agree (5) |
| Clinic leadership/managers reward innovation to improve clinical care (18) | - Strongly disagree (1) | - Somewhat disagree (2) | - Neither agree nor disagree (3) | - Somewhat agree (4) | - Strongly agree (5) |
| Clinic leadership/managers solicit opinions of clinical staff regarding decisions on patient care (19) | - Strongly disagree (1) | - Somewhat disagree (2) | - Neither agree nor disagree (3) | - Somewhat agree (4) | - Strongly agree (5) |
| Clinic leadership/managers seek ways to improve patient education and participation in care (20) | - Strongly disagree (1) | - Somewhat disagree (2) | - Neither agree nor disagree (3) | - Somewhat agree (4) | - Strongly agree (5) |

| Page Break |  |
| --- | --- |

End of Block: Inner settings

Start of Block: CME preferences

Q20.1 From which of the following CME providers did you obtain CME credit in the last 2 years? ***Please select all that apply***

- CATIE (4)
- UptoDate (5)
- Medscape (6)
- Clinical Care Options (7)
- Local CME academic organization (11)
- OCFP (10)
- College of Family Physicians of Canada (8)
- Other (9) __________________________________________________

| Page Break |  |
| --- | --- |

Q20.2 In the next 12 months, how likely  is it that you will obtain CME credits through each of the following?

|  |  |  |  |  |  |
| --- | --- | --- | --- | --- | --- |
| In-person activity (1) | - Extremely unlikely (1) | - Somewhat unlikely (2) | - Neither likely nor unlikely (3) | - Somewhat likely (4) | - Extremely likely (5) |
| Online/web-based activities (5) | - Extremely unlikely (1) | - Somewhat unlikely (2) | - Neither likely nor unlikely (3) | - Somewhat likely (4) | - Extremely likely (5) |
| Print/journal-based CME (6) | - Extremely unlikely (1) | - Somewhat unlikely (2) | - Neither likely nor unlikely (3) | - Somewhat likely (4) | - Extremely likely (5) |
| Performance improvement CME (7) | - Extremely unlikely (1) | - Somewhat unlikely (2) | - Neither likely nor unlikely (3) | - Somewhat likely (4) | - Extremely likely (5) |
| Internet Point-of-Care, e.g., Up-To-Date (8) | - Extremely unlikely (1) | - Somewhat unlikely (2) | - Neither likely nor unlikely (3) | - Somewhat likely (4) | - Extremely likely (5) |
| National/regional society meetings (9) | - Extremely unlikely (1) | - Somewhat unlikely (2) | - Neither likely nor unlikely (3) | - Somewhat likely (4) | - Extremely likely (5) |

| Page Break |  |
| --- | --- |

Q20.3 I prefer a CME that:

|  |  |  |  |  |  |
| --- | --- | --- | --- | --- | --- |
| Limits time away from practice (1) | - Not at all important (1) | - Slightly important (2) | - Moderately important (3) | - Very important (4) | - Extremely important (5) |
| Offers self-pace learning (4) | - Not at all important (1) | - Slightly important (2) | - Moderately important (3) | - Very important (4) | - Extremely important (5) |
| Is interactive with colleagues (5) | - Not at all important (1) | - Slightly important (2) | - Moderately important (3) | - Very important (4) | - Extremely important (5) |
| Is inexpensive (6) | - Not at all important (1) | - Slightly important (2) | - Moderately important (3) | - Very important (4) | - Extremely important (5) |
| Allows Interaction with colleagues/peers (8) | - Not at all important (1) | - Slightly important (2) | - Moderately important (3) | - Very important (4) | - Extremely important (5) |
| Allows interaction with course faculty (9) | - Not at all important (1) | - Slightly important (2) | - Moderately important (3) | - Very important (4) | - Extremely important (5) |
| Includes online work (10) | - Not at all important (1) | - Slightly important (2) | - Moderately important (3) | - Very important (4) | - Extremely important (5) |
| Occurs at a travel destination/spot (11) | - Not at all important (1) | - Slightly important (2) | - Moderately important (3) | - Very important (4) | - Extremely important (5) |
| Delivers activities at my practice site (12) | - Not at all important (1) | - Slightly important (2) | - Moderately important (3) | - Very important (4) | - Extremely important (5) |

End of Block: CME preferences

Start of Block: continuing education

Q21.1 In the next 12 months, how likely  is it that you will do continuing professional education through each of the following?

|  |  |  |  |  |  |
| --- | --- | --- | --- | --- | --- |
| In-person activity (1) | - Extremely unlikely (1) | - Somewhat unlikely (2) | - Neither likely nor unlikely (3) | - Somewhat likely (4) | - Extremely likely (5) |
| Online/web-based activities (5) | - Extremely unlikely (1) | - Somewhat unlikely (2) | - Neither likely nor unlikely (3) | - Somewhat likely (4) | - Extremely likely (5) |
| Print/journal-based professional education (6) | - Extremely unlikely (1) | - Somewhat unlikely (2) | - Neither likely nor unlikely (3) | - Somewhat likely (4) | - Extremely likely (5) |
| Performance improvement education (7) | - Extremely unlikely (1) | - Somewhat unlikely (2) | - Neither likely nor unlikely (3) | - Somewhat likely (4) | - Extremely likely (5) |
| Internet Point-of-Care (8) | - Extremely unlikely (1) | - Somewhat unlikely (2) | - Neither likely nor unlikely (3) | - Somewhat likely (4) | - Extremely likely (5) |
| National/regional society meetings (9) | - Extremely unlikely (1) | - Somewhat unlikely (2) | - Neither likely nor unlikely (3) | - Somewhat likely (4) | - Extremely likely (5) |

| Page Break |  |
| --- | --- |

Q21.2 I prefer a continuing educational program that:

|  |  |  |  |  |  |
| --- | --- | --- | --- | --- | --- |
| Limits time away from practice (1) | - Not at all important (1) | - Slightly important (2) | - Moderately important (3) | - Very important (4) | - Extremely important (5) |
| Offers self-pace learning (4) | - Not at all important (1) | - Slightly important (2) | - Moderately important (3) | - Very important (4) | - Extremely important (5) |
| Is interactive with colleagues (5) | - Not at all important (1) | - Slightly important (2) | - Moderately important (3) | - Very important (4) | - Extremely important (5) |
| Is inexpensive (6) | - Not at all important (1) | - Slightly important (2) | - Moderately important (3) | - Very important (4) | - Extremely important (5) |
| Allows Interaction with colleagues/peers (8) | - Not at all important (1) | - Slightly important (2) | - Moderately important (3) | - Very important (4) | - Extremely important (5) |
| Allow interaction with course faculty (9) | - Not at all important (1) | - Slightly important (2) | - Moderately important (3) | - Very important (4) | - Extremely important (5) |
| Includes online work (10) | - Not at all important (1) | - Slightly important (2) | - Moderately important (3) | - Very important (4) | - Extremely important (5) |
| Occurs at a travel destination/spot (11) | - Not at all important (1) | - Slightly important (2) | - Moderately important (3) | - Very important (4) | - Extremely important (5) |
| Delivers activities at my practice site (12) | - Not at all important (1) | - Slightly important (2) | - Moderately important (3) | - Very important (4) | - Extremely important (5) |

End of Block: continuing education

Start of Block: demographics

| Page Break |  |
| --- | --- |

Q22.1 What is your age?

- Less than 25 years old (4)
- Between 25 and 34 years old (5)
- Between 35 and 44 years old (6)
- Between 45 and 54 years old (7)
- Between 55 and 64 years old (8)
- 65 and over (9)

Q22.2 What biological sex were you assigned at birth?

- Male (1)
- Female (2)
- Intersex (3)
- Prefer not to answer (4)

Q22.3 Which of the follow apply to you? I am...

- Male/ Cisgender male (1)
- Female/ cisgender female (2)
- Gay (3)
- Bisexual (4)
- Lesbian (5)
- Transgender man (6)
- Binary (11)
- Transgender woman (7)
- Queer (8)
- Other (9)
- Prefer not to answer (10)

Q22.4 Do you consider yourself a part of (mark all that apply)

- Racial minority (1)
- Sexual minority (2)
- Religious minority (3)
- Language minority (4)

End of Block: demographics
