# Supplementary material for: Determinants of Familiarity and Experience with HIV Pre-Exposure Prophylaxis in Primary Care Providers in Ontario, Canada
Source: J Prim Care Community Health. 2025 Jan 23;16:21501319251315566. doi: 10.1177/21501319251315566 (PMC11755537; doi:10.1177/21501319251315566)
Supplement: sj-docx-2-jpc-10.1177_21501319251315566 – Supplemental material for Determinants of Familiarity and Experience with HIV Pre-Exposure Prophylaxis in Primary Care Providers in Ontario, Canada [file sj-docx-2-jpc-10.1177_21501319251315566.docx]

**Table S1. Distribution of CFIR statements related to: characteristics of PrEP and population needs**

| **Item** | **How much do you agree or disagree with the following statements** | **Strongly disagree (1),** | **Slightly disagree (2)** | **Neither agree nor disagree (3)** | **Slightly agree (4)** | **Strongly agree (5)** |
| --- | --- | --- | --- | --- | --- | --- |
| 1 | **CHARACTERISTICS OF PREP** | % | % | % | % | % |
| a1 | PrEP prevents the transmission of HIV | 1.75 |  | 1.75 | 26.32 | 70.18 |
| *a2* | *PrEP has the potential to do more harm than good if not carefully implemented* | *14.0* | *38.6* | *26.32* | *19.3* | *1.75* |
| a3 | The use of PrEP is well supported by evidence | 3.51 |  | 14.04 | 29.8 | 52.6 |
| a4 | PrEP is an effective prevention method and should be widely used as soon as possible | 1.75 | 1.75 | 10.53 | 33.3 | 52.6 |
| *a5* | *PrEP can lead to the medicalization of HIV prevention and take resources that can be better used somewhere else* | *43.8* | *24.5* | *17.5* | *10.53* | *3.51* |
| a6 | I think that PrEP is a good addition to improve the sexual health of populations at risk - | 3.51 |  | 5.26 | 19.3 | 71.9 |
| *a7* | *I think there are better alternatives than PrEP for HIV prevention -* | *14.0* | *47.3* | *33.3* | *3.53* | *1.75* |
| a8 | I think PrEP is less costly than HIV treatment | 8.77 | 1.75 | 17.5 | 31.5 | 40.3 |
| a9 | I think PrEP would be a good addition to a1condom use programs | 1.75 | 1.75 | 17.5 | 33.3 | 45.6 |
| a10 | PrEP services will be easy to implement in the clinic where I practice- | 7.0 | 26.3 | 22.8 | 19.3 | 24.5 |

**Table S2. Distribution of CFIR statements related to: characteristics of PrEP and population needs**

| **Item** | **How much do you agree or disagree with the following statements** | **Strongly disagree (1)** | **Slightly disagree (2)** | **Neither agree nor disagree (3)** | **Slightly agree (4)** | **Strongly agree (5)** |
| --- | --- | --- | --- | --- | --- | --- |
| **2a** | **POPULATION NEEDS** | % | % | % | % | % |
| *P1* | *I see very few people who may be interested in or potentially benefit from PrEP* | *5.36* | *23.2* | *19.6* | *37.5* | *14.2* |
| *P2* | *I do not think that the population I see in my clinic can afford PrEP* | *7.14* | *30.3* | *37.5* | *19.6* | *5.4* |
| *P3* | *If PrEP were implemented in the clinic where I practice, this would bother some of my patients or the local public* | *58.9* | *30.3* | *10.7* | *-* | *-* |
| *P4* | *PrEP is not a high need in the area where I practice* | *16.0* | *23.2* | *23.2* | *33.9* | *3.5* |
| *P5* | *I do not believe that the population at risk of HIV is interested in using PrEP* | *33.9* | *42.8* | *16.1* | *5.36* | *1.79* |
| *P6* | *I believe it is unethical to prescribe antiretrovirals to HIV negative people* | *75.0* | *19.7* | *5.36* |  |  |
| P7 | PrEP is something that my patients want | 1.79 | 8.9 | 51.7 | 33.9 | 3.5 |
| P8 | It is possible to set up PrEP in my practice | 7.1 | 7.1 | 16.1 | 41.1 | 28.6 |
| P9 | There is strong buy-in from community organizations for PrEP - | 3.6 | 8.9 | 46.4 | 23.2 | 17.8 |
| P10 | PrEP is something people at risk of HIV want | 1.79 |  | 21.4 | 39.3 | 37.5 |
| P11 | PrEP is something that people who receive care in my clinic want | 1.79 | 7.14 | 50.0 | 37.5 | 3.57 |
| P12 | It is possible to adapt PrEP protocols to the needs of populations at risk of HIV | 1.79 | 3.57 | 23.2 | 46.4 | 25.0 |
| P13 | It is possible to set up PrEP in the clinic where I practice | 3.64 | 7.3 | 23.7 | 40.0 | 25.5 |

**Table S3. Distribution of CFIR statements related to: concerns about PrEP**

| **2b** | As a result of the use of PrEP in populations at risk, how concerned are you about?, from to 10, with 10 highest concern | **Mean, SD** |
| --- | --- | --- |
| C1 | Developing resistance to HIV antiviral medications | 3.61; 2.35 |
| C2 | Adherence to PrEP medications | 5.22; 2.37 |
| C3 | Lower use of condoms | 4.46;3,11 |
| C4 | Misuse of medications, e.g selling medications | 1.86; 2.36 |
| C5 | Increase in sexual transmitted diseases other than HIV | 3.84;2.97 |
| C6 | PrEP related stigma | 2.81;2.68 |
| C7 | Unequal access to PrEP in some vulnerable population groups | 6.16;3.12 |
| C8 | Cost of PrEP medications | 5.77;3.12 |
| C9 | Coverage for PrEP medications | 6.48;3.01 |
| C10 | Difficulties in monitoring patients who take PrEP | 5.09;2.89 |

**Table S4. Distribution of CFIR statements related to inner setting**

|  | How much you agree or \|  disagree with the following \|  aspects of PrEP? | **Strongly disagree** | **Slightly disagree** | **Neither agree nor disagree** | **Slightly agree** | **Strongly agree** |
| --- | --- | --- | --- | --- | --- | --- |
| ***3a*** | ***COMPATIBILITY*** | % | % | % | % | % |
| *R1* | *PrEP is safe, but we are not ready to prescribe it in the clinic where I work* | *21.9* | *21.9* | *26.8* | *14.6* | *14.6* |
| R3 | It is more suitable to provide PrEP in sexual health/HIV clinics than in general primary care clinics | 19.5 | 26.8 | 29.3 | 21.9 | 2.4 |
| R4 | It is more suitable to provide PrEP in PrEP-dedicated clinics than in general primary care clinics | 21.9 | 29.3 | 26.8 | 21.9 | -- |
| R5 | I think that the prescription of PrEP should be part of the work in the clinic | 2.4 | 4.8 | 26.8 | 26.8 | 39.0 |
| **3b** | **ORGANIZATIONAL CLIMATE** | **Strongly disagree** | **Slightly disagree** | **Neither agree nor disagree** | **Slightly agree** | **Strongly agree** |
| R2 | Many colleagues around me would approve the use of PrEP | - | 2.44 | 4.88 | 41.5 | 51.2 |
| R6 | My organization/clinic is sufficiently oriented towards prevention | 3.33 | 13.3 | 6.67 | 56.7 | 20.0 |
| R7 | In the organization/clinic where I work, professionals can collaborate to offer PrEP |  | 26.9 | 23.1 | 3.8 | 19.2 |
| **3c** | **AVAILABLE RESOURCES** | **Strongly disagree** | **Slightly disagree** | **Neither agree nor disagree** | **Slightly agree** | **Strongly agree** |
| R8 | In the organization/clinic where I work, we see a good number of potential PrEP candidates | 16.7 | 25.0 | 25.0 | 29.2 | 4.2 |
| R9 | In the organization/clinic where I work, there is enough time to deliver PrEP services | 17.4 | 26.1 | 3.4 | 13.0 | 13.0 |
| R10 | In the organization/clinic where I work, there are enough resources to deliver PrEP | 3.8 | 30.8 | 27 | 15.4 | 23.1 |
| R11 | My clinic has the necessary resources and support to set up PrEP services | 12.5 | 16.7 | 29.2 | 25 | 16.7 |
| R12 | My clinic has the necessary staffing to support the implementation of PrEP | 7.14 | 28.6 | 17.9 | 32.1 | 14.3 |

**Table S5. Distribution of CFIR statements related to inner setting**

| **3d** | **LEADERSHIP ENGAGEMENT** | **Strongly disagree** | **Slightly disagree** | **Neither agree nor disagree** | **Slightly agree** | **Strongly agree** |
| --- | --- | --- | --- | --- | --- | --- |
|  |  | % | % | % | % | % |
| R13 | Clinic leadership/managers reward innovation to improve clinical care | 12.5 | 12.5 | 37.5 | 33.3 | 4.17 |
| R14 | Clinic leadership/managers solicit opinions of clinical staff regarding decisions on patient care | -- | 9.1 | 31.9 | 36.4 | 22.7 |
| R15 | Clinic leadership/managers seek ways to improve patient education and participation in care | -- | 5.2 | 52.6 | 31.5 | 10.5 |

**Table S6 . Distribution of CFRI statements related to level of comfort with clinical practices**

| **4a** | **How would you describe your comfort level with the following clinical practices?** | Completely comfortable | Very comfortable | Somewhat comfortable | Slightly comfortable | Not at all comfortable |
| --- | --- | --- | --- | --- | --- | --- |
|  |  | % | % | % | % | % |
| S1 | Inquiring about sexual orientation | 44.4 | 31.5 | 16.7 | 7.4 | - |
| S2 | Discussing sexual habits with your patients | 37.1 | 35.2 | 18.5 | 9.26 | - |
| S3 | HIV screening | 42.5 | 35.2 | 14.8 | 3.7 | 3.7 |
| S4 | Screening for high-risk sexual habits | 35.2 | 27.8 | 25.9 | 11.1 | - |
| S5 | Providing risk reduction counseling | 27.8 | 38.9 | 18.6 | 14.8 | - |
| S6 | Ordering a diagnostic test for acute HIV | 45.1 | 21.6 | 21.6 | 9.8 | 1.96 |
| S7 | Revealing an HIV diagnosis to your patient(s) | 16.9 | 22.6 | 33.9 | 13.2 | 13.2 |
| S8 | Managing HIV medications | 6.12 | 4.1 | 12.2 | 20.4 | 57.1 |
| S9 | Ordering tests for sexually transmitted infections (Chlamydia, syphilis, gonorrhea) | 63.4 | 32.7 | 3.8 | - | - |

**Table S7. Distribution of CFIR statements related to Self-efficacy with PrEP practices**

| **4b** | **On a scale from 1 to 10, how confident are you that you are ABLE to perform the following tasks?, where 10 is the max ability** | Mean, SD, max 10 |
| --- | --- | --- |
| SE1 | Offering complete PrEP care | 3.77;3.04 |
| SE2 | Counselling patients about PrEP | 4.7; 3.22 |
| SE3 | Using the tools to identify patients eligible for PrEP | 4.68; 3.27 |
| SE4 | Offering PrEP care if supported with a clear protocol and prescribing tools | 6.3; 3.4 |
| SE5 | Managing effects of medications used for PrEP | 4.4;3.03 |

**Table S8. Distribution of CFIR statements related to individual characteristics: compatibility, beliefs about consequences**

|  | **1 How much do you agree or disagree with the following statements** | **Strongly disagree** | **Slightly disagree** | **Neither agree nor disagree** | **Slightly agree** | **Strongly agree** |
| --- | --- | --- | --- | --- | --- | --- |
| **4c** | ***COMPATIBILITY*** | % | % | % | % | % |
| B1 | I see my values reflected in the implementation of PrEP | 5.36 | 1.79 | 19.6 | 39.3 | 33.9 |
| B2 | PrEP will be a very good fit in my clinic (3) | 5.36 | 8.93 | 30.4 | 32.1 | 23.2 |
| B3 | Managing PrEP will be compatible with my work in the clinic (4) | 5.36 | 8.9 | 25 | 41.1 | 19.7 |
| B4 | I see my personal values reflected in the implementation of PrEP ( | 5.36 | 3.57 | 19.6 | 35.7 | 35.7 |
| B5 | It is my responsibility as a primary care provider to offer PrEP services to patients who may need it | 3.57 | 1.79 | 8.93 | 26.8 | 58.9 |
| B6 | I think that managing PrEP patients will be worth it | 3.57 | - | 17.9 | 35.7 | 42.8 |
| B7 | Prescribing and/or managing PrEP should be part of the work I do in the clinic | 7.14 | 3.5 | 19.7 | 35.7 | 33.9 |
| *B8* | *For me, managing PrEP will not be useful at all* | 42.8 | 25 | 23.2 | 5.36 | 3.6 |
| *B9* | *Managing PrEP is not a priority for me* | 28.6 | 25 | 25 | 19.7 | 1.8 |

**Table S9. Distribution of CFIR statements related to individual characteristics: compatibility, beliefs about consequences**

|  | **1 How much do you agree or disagree with the following statements** | **Strongly disagree** | **Slightly disagree** | **Neither agree nor disagree** | **Slightly agree** | **Strongly agree** |
| --- | --- | --- | --- | --- | --- | --- |
| **4d** | ***CONSEQUENCES*** | % | % | % | % | % |
| B10 | I can bill for managing PrEP patients | 4.76 | 2.38 | 28.6 | 28.6 | 35.7 |
| B11 | If I manage PrEP patients, I can obtain recognition from my colleagues | 6.67 | 6.67 | 62.2 | 24.4 | -- |
| B12 | If I manage PrEP patients, I will help my patients to reduce their risk of HIV | -- | -- | 4.0 | 22.0 | 74.0 |
| B13 | If I manage PrEP patients, I will get recognition from my patients | 6.12 | 2.04 | 36.7 | 42.8 | 12.2 |
| B14 | If I manage PrEP patients, this will strengthen collaboration with other professionals | 4.1 | 10.2 | 28.6 | 40.8 | 16.3 |
| B15 | If I manage PrEP patients, I will get recognition in my organization | 8.7 | 10.9 | 58.7 | 21.7 | -- |
| B16 | Most people who are important to me think that I should manage PrEP patients | 4.6 | 11.6 | 55.8 | 18.6 | 9.3 |
| B17 | By managing PrEP, I would receive recognition from professionals that are important to me | 6.8 | 13.6 | 56.8 | 18.2 | 4.5 |
